# Advocacy Training as a Way to Increase Dementia Literacy Among College Students

**DOI:** 10.1093/geroni/igaa057.180

**Published:** 2020-12-16

**Authors:** Tsuann Kuo

**Affiliations:** Chung Shan Medical University, Taichung City, Taiwan (Republic of China)

## Abstract

In 2017, WHO passed the Global Action Plan on Dementia and declared the effort to increase dementia literacy around the world. Although dementia is mostly associated with older adults, the prevalent rate of the early on-set and the need to take care of people with dementia has mostly fallen on younger family members. Therefore, the purpose of this paper is to develop an intervention aiming to increase dementia awareness among college students. Through training and action plans, 85 university students from 9 different departments formed 12 groups to develop creatively and to conduct advocacy or public education in various communities. A questionnaire of pre- and post-test was conducted after students had completed their action plans. The response rate was 86% and the results were three-folds: (1) 31% of the students had someone in the family with dementia; (2) 35% of the students indicated that they were familiar with dementia; and (3) the pre- and post-scores on dementia awareness (p<0.001) and dementia attitudes (p<0.001) had significant improvement. This study demonstrated that there is a need to start the effort to increase dementia literacy because one-third of college-age students might be potential caregivers for their family loved ones with dementia. Preparing the students before graduating from college is a good entry because they can become health professionals to take better care of dementia patients and their family members. In conclusion, policy and practice implications will be discussed so the communities can become more dementia friendly in the future.

